# Short-term evolution strategies for host adaptation and drug escape in human fungal pathogens

**DOI:** 10.1371/journal.ppat.1008519

**Published:** 2020-05-14

**Authors:** Chapman N. Beekman, Iuliana V. Ene

**Affiliations:** Department of Molecular Microbiology and Immunology, Brown University, Providence, Rhode Island, United States of America; McGill University, CANADA

Research on human fungal pathogens has historically taken a backseat to other infectious diseases, perhaps due to a common misperception that fungi largely cause superficial infections [1]. In reality, fungi can be life-threatening to those who become immunocompromised during medical procedures or through conditions such as HIV and diabetes. Invasive fungal infections are estimated to kill over 1 million people every year, with mortality rates reaching 50% [2]. Significant challenges to the treatment of fungal infections include the limited availability of antifungals and the innate ability of fungi to rapidly evolve and adapt to fluctuating conditions. This adaptive ability is partially driven by extensive genomic plasticity, with many species acquiring diverse ploidy states, chromosomal rearrangements, and point mutations during host colonization [3–8]. Genetic plasticity enables rapid increases in virulence and antifungal drug resistance, which often translate to poor disease outcomes. Short-term evolution (microevolution) strategies in fungal pathogens are therefore essential for environmental adaptation in the mammalian host, and their study can inform adaptive mechanisms in other eukaryotes.

## Ploidy shifts enable rapid fitness jumps under stressful conditions

Many clinically relevant fungi display dynamic changes in ploidy, including both karyotypic variations (number of sets of chromosomes) as well as aneuploidy (imbalance in chromosome copy number). Some fungal pathogens exist as stable haploid, diploid, or polyploid cells, but ploidy can change upon shifting conditions. Alterations in baseline ploidy have been described for some of the most prevalent genera (*Candida*, *Cryptococcus*, and *Aspergillus*) and are often selected for in the host or during antifungal treatment. Extra chromosomes are common in isolates from human infections [5, 6, 8, 9] and after passage through mammalian hosts during experimental microevolution [10–12]. Under nutrient starvation, *Candida albicans* isolates can favor either near-haploid or near-diploid states, indicating that karyotypic reduction can provide an efficient adaptive route in some conditions [13]. Aneuploidy is also common in *C*. *albicans* and in *Cryptococcus neoformans* lineages and has been linked to increased virulence and drug resistance [14] [15]. Chromosomal duplication can mediate adaptation through gene dosage, as transcript levels are often proportional to gene copy number [16]. This can be seen in both *C*. *albicans* and *Cryptococcus* species, for which antifungal treatment selects for increased copies of chromosomes or chromosomal segments containing drug targets and/or efflux pumps. Thus, clinical isolates of *Cryptococcus* lineages VNI and VGI that persisted during fluconazole therapy were frequently disomic for chromosome 1 [5]. Analogous in vitro fluconazole treatment of *Cryptococcus* lineages VNI and VNIV selected for disomy of chromosome 1, which contains both the azole target *ERG11* and the major azole transporter, *AFR1* [12]. Similarly, in *C*. *albicans*, formation of different isochromosomes (partial duplication, i(4R) [17] and i(5L) [9]) enabled azole resistance, while trisomy of chromosome 2 conferred caspofungin resistance [18]. Together, these studies suggest that ploidy alterations are a common mechanism enabling adaptation to sudden stress including antifungal treatment.

## Loss of heterozygosity as a driver of phenotypic variation in diploid species

Another strategy that has emerged as an important mechanism for adaptation is loss of heterozygosity (LOH) or the loss of genetic information from one chromosome homolog. LOH can arise via chromosomal double-strand breaks (followed by break-induced replication or other repair mechanisms) as well as via recombination between homologous chromosomal regions, which can occur both mitotically and meiotically. LOH events can vary dramatically in size, depending on the type of event, affecting single polymorphisms (i.e., gene conversions) to whole chromosomes (i.e., non-disjunctions). Large LOH events can single-handedly impact multiple genes through the homozygosis of hundreds to thousands of positions [19]. While diploid genomes minimize the impact of de novo mutation by maintaining an ancestral copy of an allele, LOH can unmask recessive variants under conditions where they endow a fitness benefit. This is particularly relevant for heterozygous diploid *Candida* species but also for *C*. *neoformans* VNIII (AD) hybrids [20]. LOH events across short regions via gene conversion or via segmental or whole chromosome loss are common in *C*. *albicans* clinical isolates [8, 17] and strains passaged in mice [3, 4, 10, 21]. Studies of LOH distribution found the majority of LOH breakpoints within or adjacent to repeat sequences, suggesting repetitive DNA may promote genetic recombination between or within homologs [17, 19]. Such recombination events can lead to subsequent de novo mutations via error-prone DNA repair mechanisms, further adding to the ability of LOH to accelerate fungal evolution. Evidence of this phenomenon can be seen in the significant enrichment of heterozygous single nucleotide polymorphisms (SNPs) adjacent to LOH breakpoints in *C*. *albicans* [19]. LOH can also maximize the impact of de novo point mutations. Analysis of serial isolates from 11 candidiasis patients detected large LOH events in isolates from all patients, resulting in the homozygosis of 130 de novo SNPs [8]. Several *C*. *albicans* studies found that LOH on one chromosome significantly increases the likelihood of LOH at other loci, thereby amplifying the impact of LOH in this species and suggesting that LOH may be a concerted process [4, 21, 22].

A couple of recent examples highlight how LOH across recessive alleles can alter both virulence and commensalism in *C*. *albicans*. In one study, passage through the mouse gastrointestinal (GI) tract selected for LOH events, which inactivated the hyphal-regulator *FLO8* via homozygosis of nonsense and frameshift mutations [10]. Loss of Flo8 led to a commensal phenotype, with strains losing their virulence and providing immune-priming against secondary fungal or bacterial infections [10]. Loss of function of another central regulator of filamentation, Efg1, was similarly associated with increased fitness in the mouse GI tract [23]. Interestingly, heterozygous null mutations in *EFG1* are common across *C*. *albicans* clinical isolates, and frequent LOH at this locus led to *EFG1* inactivation during GI tract passage [24]. These findings demonstrate how LOH events can significantly impact the evolutionary trajectories of *C*. *albicans* during host colonization. Furthermore, LOH of hemizygous alleles of key regulators may represent a common mechanism by which *C*. *albicans* can increase its fitness in the host.

## Small-scale genetic variation with genome-wide consequences

Large-scale genomic changes such as aneuploidy and long-tract LOH events can affect hundreds to thousands of genes in a single cell cycle but also carry a significant deleterious risk. It is therefore not surprising that the majority of mutational events observed during fungal microevolution are represented by small-scale mutations, including SNPs, insertions and deletions (indels), and short-tract LOH [7, 8, 19]. These small variants can also have extensive downstream consequences when they affect critical genes. One example are mutations in central transcription factors, in which a single mutation can alter the expression of hundreds of genes. In *C*. *neoformans* VNI, the commonly used laboratory lineage H99 contains a deletion in the transcriptional regulator Sgf29 encoding a histone acetylase [25]. Several clinical isolates independently evolved loss of function mutations in the *SGF29* gene, the inactivation of which impacted acetylation at more than 700 loci and led to a hypervirulent phenotype [25].

Mutations within DNA repair genes can also impact trajectories of evolution. Mutations in the mismatch repair gene *MSH2* have been reported in *Candida glabrata* [26] and *C*. *neoformans* VNI [27], where they produce a hypermutator phenotype. In *C*. *neoformans* VNI, *MSH2* loss of function mutations produced strains with high mutation rates and extensive phenotypic variability [27]. In *C*. *neoformans* VNI and VNIV, an amino acid substitution in the DNA polymerase gene *POL3* also caused rapid microevolution without an apparent fitness cost [28]. In both species, the disruption of mismatch repair mechanisms enabled development of drug resistance, demonstrating how accelerated mutation rates can enable rapid adaptation [27] [28].

## Escape from antifungal treatment

The ability of fungal pathogens to evolve resistance within the time frame of a single infection can be life-threatening. Development of antifungal resistance is common during human infections by the major pathogens *C*. *albicans* [8], *A*. *fumigatus* [29], and *Cryptococcus* species [5]. Experimental evolution using different in vitro antifungal treatments have further demonstrated the ability of fungal pathogens to rapidly acquire drug resistance [12, 21, 30]. Some studies indicate that acquisition of drug resistance may carry a fitness cost in vitro, as resistance is often lost during subsequent passage in the absence of drug [5, 31]. However, the impact of drug resistance on fitness within the host is more complex as resistant isolates can arise in patients even without antifungal treatment via mutations that provide protection against chemically-similar compounds [32]. This is seen in *Candida lusitaniae* lung isolates for which mutations in the *MRR1* transcription factor gene confer azole resistance and also contribute to resistance against the host antimicrobial peptide histatin 5 and bacterial phenazines [32].

The types of genetic mutations underlying antifungal resistance are diverse across species and drugs. As discussed above, both *Candida* [9, 30] and *Cryptococcus* [5, 12] utilize aneuploidy and associated increases in gene expression of antifungal targets and efflux pumps to escape drug treatment. In *Candida* species, single base pair mutations in key cellular efflux regulators *MRR1* [32], *TAC1* [33], or *PDR1* [34] have been linked to multidrug resistance. LOH can further contribute to this process by enabling homozygosis of the mutated alleles, as observed for both *MRR1* [32] and *TAC1* [33] genes. Point mutations within drug target genes themselves can also drive resistance in *Candida* species, as in the case of β-glucan synthase genes *FKS1* and *FKS2* (targets of echinocandins) [35, 36] and ergosterol pathway gene *ERG11* (target of azoles) [37]. In *Aspergillus*, similar mutations in *CYP51* (the *ERG11* homolog) reduce the affinity between azole and drug target, thereby leading to drug resistance [38]. In addition to providing a strong selective pressure, exposure to antifungals may themselves promote genetic variation. This is seen in *C*. *albicans*, in which antifungal treatment results in increased genome instability and higher frequencies of LOH and point mutations [22, 39]. Antifungals therefore drive fungal microevolution by both increasing the rates of genetic variation and by selecting for tolerant and/or resistant isolates.

## Microevolution in complex host environments

In contrast to the defined environments in which fungi are passaged in the laboratory, selective pressures encountered in the host are complex and multifaceted. Here, commensal and pathogenic fungi must contend with constant host immune surveillance, fluctuating pH and nutrient availability, as well as compete with resident microbes. This array of selective pressures results in a wider range of genetic variability than typically seen following in vitro passage. Indeed, for *C*. *albicans*, higher numbers of mutations and rates of recombination were estimated to occur during passage in the host (bloodstream infection and GI tract colonization) relative to growth in rich laboratory media [4, 19]. While it is extremely challenging to evaluate the relative contribution of individual selective forces, mutations accumulated within the host can provide important clues. In *C*. *lusitaniae*, mutations in *MRR1*, which endow azole resistance, also protect against histastin-5, an antimicrobial peptide of the innate immune system [32]. Alterations in cell wall–related genes were also common in serial clinical isolates of this species obtained from blood and lung samples [6]. Similarly, *C*. *albicans* passage through bloodstream and GI mouse infection models resulted in frequent mutations in cell wall adhesin genes relative to other coding regions [19], perhaps due to the large number of tandem repeats that these genes carry. Passage of a yeast-locked *C*. *albicans* strain within murine macrophages led to restored filamentation and virulence via a single nucleotide change in the Mediator component *SSN3*, thereby illustrating that complex regulatory networks can be easily rewired through microevolution [40]. Together, these studies indicate that the host immune system is a key driver of microevolution and the cell wall surface is likely an important site of genetic variation.

In addition to host immunity, host-resident microbes also exert substantial pressure on fungal pathogens. In fact, GI passage of *C*. *albicans* within immunodeficient and antibiotic treated neonatal mice showed that adaptation to this niche was dependent upon inhibition of the microbiota, suggesting that host microbes limit the adaptation of this commensal species to the mouse GI [10]. Metabolic pressures within the host may also drive fungal microevolution. In *C*. *neoformans* VNI, serial passage through the mouse brain resulted in a mutant with increased expression of an iron reductase [41]. Host-mediated nutritional immunity of micronutrients such as iron and zinc (reviewed in [42]) is likely an important selective pressure in diverse host environments. *C*. *albicans* strains passaged in the mouse GI repeatedly evolved trisomies for chromosome 7, which resulted in increased fitness for this niche [19]. This aneuploidy was later implicated in susceptibility to medium chain fatty acids [43]. Oxidative metabolism may also be under strong selection within the host. In one example, passage of *C*. *albicans* through a systemic infection model yielded a respiration-deficient strain, which was resistant to phagocytosis and proliferated in the host with decreased morbidity [44]. In *A*. *fumigatus*, fitness in low oxygen was associated with higher virulence in a murine lung infection model [45]. Further investigation revealed that adaptation to low oxygen in this species resulted in altered growth morphology, cell wall architecture, and hyphal adhesion, which in turn increased virulence via immunopathological inflammation [46]. These studies illustrate the complexity of fungal microevolution in the host environment, demonstrating how seemingly unrelated processes such as metabolic adaptation and immune evasion can be linked.

## Balancing novel genetic variation, genome integrity, and fitness trade-offs

Human fungal pathogens utilize a broad array of strategies to generate genetic diversity and adapt to fluctuating environments. This genomic flexibility enables rapid adaptation to antifungal drugs or selective pressures within the host. While the ability of fungi to tolerate significant genomic plasticity provides increased adaptability, this raises the question of how this ability is balanced against the need to maintain genome integrity. This requirement may be relaxed in host niches with limited timescales due to rapid host death or those that do not facilitate pathogen transmission (e.g., deep-seated infections). However, genome integrity is crucial for fungal viability in host niches with sustained colonization such as skin, lungs, genitourinary, and GI tracts.

**Fig 1:**
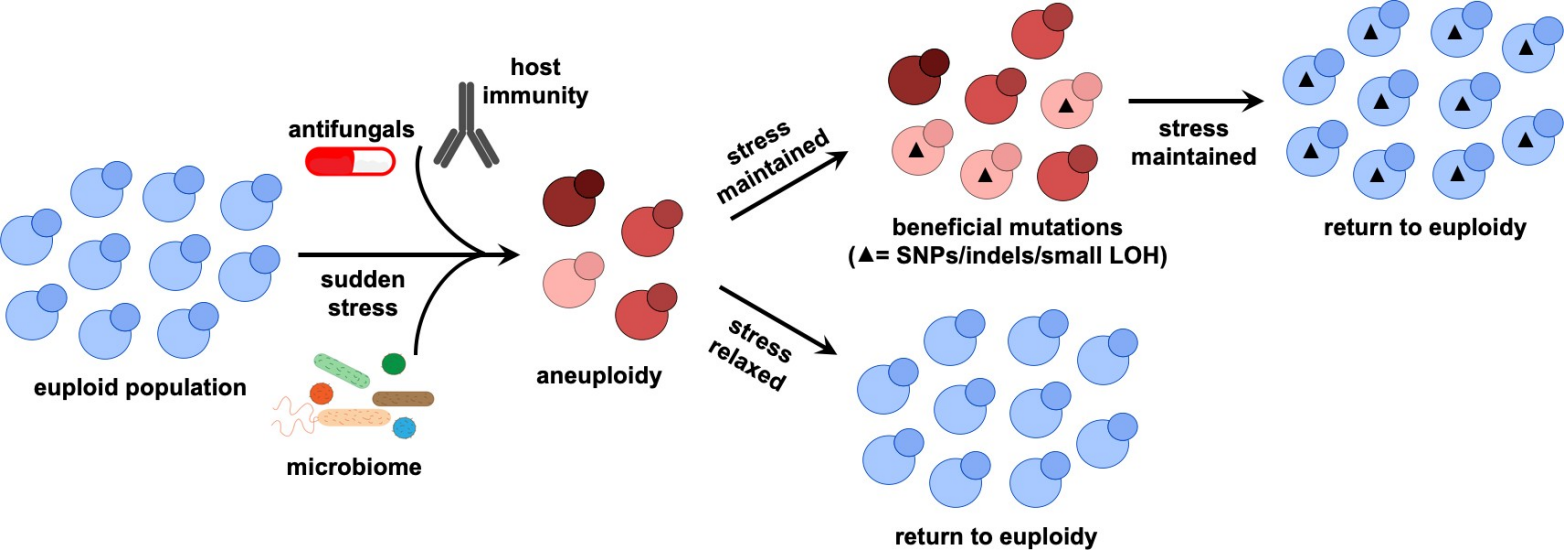
Hypothetical model for how aneuploidy can be a transient solution to stress adaptation. Stressful environments can trigger the formation of aneuploid isolates. Once selective pressures are relieved or beneficial mutations with smaller fitness costs are acquired, cells may return to the euploid state. Different red-colored cells represent cells with different ploidy levels and black triangles represent beneficial mutations. LOH, loss of heterozygosity.

The need to maintain genome integrity becomes even more pressing when considering that aneuploidy and LOH are associated with a global increase in genome instability [4, 21, 47]. Aneuploidy is often unstable in the absence of selection [12, 31] and is associated with delayed cell division and increased proteotoxic, oxidative, and hypo-osmotic stress [48, 49], therefore additional fitness costs may be incurred. To mitigate these fitness costs, fungi may employ chromosomal rearrangements only as a temporary solution to sudden stress, thus “buying time” to acquire less costly mutations such as SNPs and indels [50] (Fig 1). However, how these species balance genomic plasticity with genomic integrity remains a large gap in our understanding of fungal pathogens.

The need to better understand fungal microevolution is emphasized by our inability to effectively treat invasive fungal infections, a major challenge given the limited repertoire of antifungals available. A better understanding of the relationship between commensalism and virulence is also fundamental. Given the diversity of host niches that fungi can occupy, microevolution can rapidly tilt the balance between commensalism and pathogenesis. This is demonstrated in *C*. *albicans* in which strains adapted to the mouse GI [10, 24] or oropharyngeal tracts [11] lost the ability to cause lethal systemic infection. Conversely, isolates recovered from kidneys during serial systemic infection display accelerated host killing [51]. Understanding the selective pressures within specific host niches and how fungal genomes respond to them during microevolution is central to defining the mechanisms by which fungi adapt and evolve, particularly in the context of mammalian host infection.

## References

[1] Stop neglecting fungi. Nat Microbiol. 2017;2:17120 Epub 2017/07/26. 10.1038/nmicrobiol.2017.120 . 28741610

[2] Brown GD, Denning DW, Gow NA, Levitz SM, Netea MG, White TC. Hidden killers: human fungal infections. Sci Transl Med. 2012;4(165):165rv13. Epub 2012/12/21. 10.1126/scitranslmed.3004404 . 23253612

[3] Forche A, Cromie G, Gerstein AC, Solis NV, Pisithkul T, Srifa W, et al Rapid Phenotypic and Genotypic Diversification After Exposure to the Oral Host Niche in *Candida albicans*. Genetics. 2018;209(3):725–41. Epub 2018/05/05. 10.1534/genetics.118.301019 29724862 PMC6028260

[4] Forche A, Magee PT, Selmecki A, Berman J, May G. Evolution in *Candida albicans* populations during a single passage through a mouse host. Genetics. 2009;182(3):799–811. Epub 2009/05/06. 10.1534/genetics.109.103325 19414562 PMC2710160

[5] Stone NR, Rhodes J, Fisher MC, Mfinanga S, Kivuyo S, Rugemalila J, et al Dynamic ploidy changes drive fluconazole resistance in human *cryptococcal* meningitis. J Clin Invest. 2019;129(3):999–1014. Epub 2019/01/29. 10.1172/JCI124516 30688656 PMC6391087

[6] Carrete L, Ksiezopolska E, Gomez-Molero E, Angoulvant A, Bader O, Fairhead C, et al Genome Comparisons of *Candida glabrata* Serial Clinical Isolates Reveal Patterns of Genetic Variation in Infecting Clonal Populations. Front Microbiol. 2019;10:112 Epub 2019/02/28. 10.3389/fmicb.2019.00112 30809200 PMC6379656

[7] Ballard E, Melchers WJG, Zoll J, Brown AJP, Verweij PE, Warris A. In-host microevolution of *Aspergillus fumigatus*: A phenotypic and genotypic analysis. Fungal Genet Biol. 2018;113:1–13. Epub 2018/02/27. 10.1016/j.fgb.2018.02.003 29477713 PMC5883321

[8] Ford CB, Funt JM, Abbey D, Issi L, Guiducci C, Martinez DA, et al The evolution of drug resistance in clinical isolates of *Candida albicans*. Elife. 2015;4:e00662 Epub 2015/02/04. 10.7554/eLife.00662 25646566 PMC4383195

[9] Selmecki A, Forche A, Berman J. Aneuploidy and isochromosome formation in drug-resistant *Candida albicans*. Science. 2006;313(5785):367–70. Epub 2006/07/22. 10.1126/science.1128242 16857942 PMC1717021

[10] Tso GHW, Reales-Calderon JA, Tan ASM, Sem X, Le GTT, Tan TG, et al Experimental evolution of a fungal pathogen into a gut symbiont. Science. 2018;362(6414):589–95. Epub 2018/11/06. 10.1126/science.aat0537 . 30385579

[11] Forche A, Solis NV, Swidergall M, Thomas R, Guyer A, Beach A, et al Selection of *Candida albicans* trisomy during oropharyngeal infection results in a commensal-like phenotype. PLoS Genet. 2019;15(5):e1008137. Epub 2019/05/16. doi: 10.1371/journal.pgen.1008137 PubMed PMID: 31091232; PubMed Central PMCID: PMC6538192. 31091232 PMC6538192

[12] Sionov E, Lee H, Chang YC, Kwon-Chung KJ. *Cryptococcus neoformans* overcomes stress of azole drugs by formation of disomy in specific multiple chromosomes. PLoS Pathog. 2010;6(4):e1000848 Epub 2010/04/07. 10.1371/journal.ppat.1000848 20368972 PMC2848560

[13] Gerstein AC, Lim H, Berman J, Hickman MA. Ploidy tug-of-war: Evolutionary and genetic environments influence the rate of ploidy drive in a human fungal pathogen. Evolution. 2017;71(4):1025–38. Epub 2017/02/15. 10.1111/evo.13205 . 28195309 PMC7035954

[14] Hickman MA, Paulson C, Dudley A, Berman J. Parasexual Ploidy Reduction Drives Population Heterogeneity Through Random and Transient Aneuploidy in *Candida albicans*. Genetics. 2015;200(3):781–94. Epub 2015/05/21. 10.1534/genetics.115.178020 25991822 PMC4512543

[15] Morrow CA, Fraser JA. Ploidy variation as an adaptive mechanism in human pathogenic fungi. Semin Cell Dev Biol. 2013;24(4):339–46. Epub 2013/02/06. 10.1016/j.semcdb.2013.01.008 . 23380396

[16] Tucker C, Bhattacharya S, Wakabayashi H, Bellaousov S, Kravets A, Welle SL, et al Transcriptional Regulation on Aneuploid Chromosomes in Divers *Candida albicans* Mutants. Sci Rep. 2018;8(1):1630 Epub 2018/01/28. 10.1038/s41598-018-20106-9 29374238 PMC5786073

[17] Todd RT, Wikoff TD, Forche A, Selmecki A. Genome plasticity in *Candida albicans* is driven by long repeat sequences. Elife. 2019;8 Epub 2019/06/08. 10.7554/eLife.45954 31172944 PMC6591007

[18] Yang F, Teoh F, Tan ASM, Cao Y, Pavelka N, Berman J. Aneuploidy Enables Cross-Adaptation to Unrelated Drugs. Mol Biol Evol. 2019;36(8):1768–82. Epub 2019/04/28. 10.1093/molbev/msz104 31028698 PMC6657732

[19] Ene IV, Farrer RA, Hirakawa MP, Agwamba K, Cuomo CA, Bennett RJ. Global analysis of mutations driving microevolution of a heterozygous diploid fungal pathogen. Proc Natl Acad Sci U S A. 2018;115(37):E8688–E97. Epub 2018/08/29. 10.1073/pnas.1806002115 30150418 PMC6140516

[20] Hu G, Liu I, Sham A, Stajich JE, Dietrich FS, Kronstad JW. Comparative hybridization reveals extensive genome variation in the AIDS-associated pathogen *Cryptococcus neoformans*. Genome Biol. 2008;9(2):R41 Epub 2008/02/26. 10.1186/gb-2008-9-2-r41 18294377 PMC2374700

[21] Popp C, Ramirez-Zavala B, Schwanfelder S, Kruger I, Morschhauser J. Evolution of Fluconazole-Resistant Candida albicans Strains by Drug-Induced Mating Competence and Parasexual Recombination. MBio. 2019;10(1). Epub 2019/02/07. 10.1128/mBio.02740-18 30723130 PMC6428756

[22] Forche A, Abbey D, Pisithkul T, Weinzierl MA, Ringstrom T, Bruck D, et al Stress alters rates and types of loss of heterozygosity in *Candida albicans*. MBio. 2011;2(4). Epub 2011/07/28. 10.1128/mBio.00129-11 21791579 PMC3143845

[23] Pande K, Chen C, Noble SM. Passage through the mammalian gut triggers a phenotypic switch that promotes *Candida albicans* commensalism. Nat Genet. 2013;45(9):1088–91. Epub 2013/07/31. 10.1038/ng.2710 23892606 PMC3758371

[24] Liang SH, Anderson MZ, Hirakawa MP, Wang JM, Frazer C, Alaalm LM, et al Hemizygosity Enables a Mutational Transition Governing Fungal Virulence and Commensalism. Cell Host Microbe. 2019;25(3):418–31 e6. Epub 2019/03/03. 10.1016/j.chom.2019.01.005 30824263 PMC6624852

[25] Arras SDM, Ormerod KL, Erpf PE, Espinosa MI, Carpenter AC, Blundell RD, et al Convergent microevolution of *Cryptococcus neoformans* hypervirulence in the laboratory and the clinic. Sci Rep. 2017;7(1):17918 Epub 2017/12/22. 10.1038/s41598-017-18106-2 29263343 PMC5738413

[26] Healey KR, Zhao Y, Perez WB, Lockhart SR, Sobel JD, Farmakiotis D, et al Prevalent mutator genotype identified in fungal pathogen *Candida glabrata* promotes multi-drug resistance. Nat Commun. 2016;7:11128 Epub 2016/03/30. 10.1038/ncomms11128 27020939 PMC5603725

[27] Boyce KJ, Wang Y, Verma S, Shakya VPS, Xue C, Idnurm A. Mismatch Repair of DNA Replication Errors Contributes to Microevolution in the Pathogenic Fungus *Cryptococcus neoformans*. MBio. 2017;8(3). Epub 2017/06/01. 10.1128/mBio.00595-17 28559486 PMC5449657

[28] Boyce KJ, Cao C, Xue C, Idnurm A. A spontaneous mutation in DNA polymerase POL3 during in vitro passaging causes a hypermutator phenotype in *Cryptococcus* species. DNA Repair (Amst). 2020;86:102751 Epub 2019/12/16. 10.1016/j.dnarep.2019.102751 . 31838381 PMC7542539

[29] Mortensen KL, Jensen RH, Johansen HK, Skov M, Pressler T, Howard SJ, et al Aspergillus species and other molds in respiratory samples from patients with cystic fibrosis: a laboratory-based study with focus on *Aspergillus fumigatus* azole resistance. J Clin Microbiol. 2011;49(6):2243–51. Epub 2011/04/22. 10.1128/JCM.00213-11 21508152 PMC3122734

[30] Gerstein AC, Berman J. Diversity of acquired adaptation to fluconazole is influenced by genetic background and ancestral fitness in *Candida albicans*. bioRxiv [preprint]. 2018 BioRxiv 360347. Available from: https://www.biorxiv.org/content/10.1101/360347v4

[31] Hirakawa MP, Chyou DE, Huang D, Slan AR, Bennett RJ. Parasex Generates Phenotypic Diversity de Novo and Impacts Drug Resistance and Virulence in *Candida albicans*. Genetics. 2017;207(3):1195–211. Epub 2017/09/16. 10.1534/genetics.117.300295 28912344 PMC5676243

[32] Demers EG, Biermann AR, Masonjones S, Crocker AW, Ashare A, Stajich JE, et al Evolution of drug resistance in an antifungal-naive chronic *Candida lusitaniae* infection. Proc Natl Acad Sci U S A. 2018;115(47):12040–5. Epub 2018/11/06. 10.1073/pnas.1807698115 30389707 PMC6255150

[33] Coste A, Turner V, Ischer F, Morschhauser J, Forche A, Selmecki A, et al A mutation in Tac1p, a transcription factor regulating CDR1 and CDR2, is coupled with loss of heterozygosity at chromosome 5 to mediate antifungal resistance in *Candida albicans*. Genetics. 2006;172(4):2139–56. Epub 2006/02/03. 10.1534/genetics.105.054767 16452151 PMC1456413

[34] Vermitsky JP, Earhart KD, Smith WL, Homayouni R, Edlind TD, Rogers PD. Pdr1 regulates multidrug resistance in *Candida glabrata*: gene disruption and genome-wide expression studies. Mol Microbiol. 2006;61(3):704–22. Epub 2006/06/29. 10.1111/j.1365-2958.2006.05235.x . 16803598

[35] Garcia-Effron G, Lee S, Park S, Cleary JD, Perlin DS. Effect of *Candida glabrata FKS1* and *FKS2* mutations on echinocandin sensitivity and kinetics of 1,3-beta-D-glucan synthase: implication for the existing susceptibility breakpoint. Antimicrob Agents Chemother. 2009;53(9):3690–9. Epub 2009/06/24. 10.1128/AAC.00443-09 19546367 PMC2737881

[36] Desnos-Ollivier M, Bretagne S, Raoux D, Hoinard D, Dromer F, Dannaoui E, et al Mutations in the fks1 gene in *Candida albicans*, *C*. *tropicalis*, and *C*. *krusei* correlate with elevated caspofungin MICs uncovered in AM3 medium using the method of the European Committee on Antibiotic Susceptibility Testing. Antimicrob Agents Chemother. 2008;52(9):3092–8. Epub 2008/07/02. 10.1128/AAC.00088-08 18591282 PMC2533459

[37] Xiang MJ, Liu JY, Ni PH, Wang S, Shi C, Wei B, et al Erg11 mutations associated with azole resistance in clinical isolates of *Candida albicans*. FEMS Yeast Res. 2013;13(4):386–93. Epub 2013/03/14. 10.1111/1567-1364.12042 . 23480635

[38] Perez-Cantero A, Lopez-Fernandez L, Guarro-Artigas J, Capilla J. Update and recent advances on azole resistance mechanisms in *Aspergillus*. Int J Antimicrob Agents. 2019 Epub 2019/09/23. 10.1016/j.ijantimicag.2019.09.011 PubMed PMID: 31542320. 31542320

[39] Avramovska O, Hickman MA. The Magnitude of *Candida albicans* Stress-Induced Genome Instability Results from an Interaction Between Ploidy and Antifungal Drugs. G3 (Bethesda). 2019 Epub 2019/10/06. 10.1534/g3.119.400752 . 31585926 PMC6893200

[40] Wartenberg A, Linde J, Martin R, Schreiner M, Horn F, Jacobsen ID, et al Microevolution of *Candida albicans* in macrophages restores filamentation in a nonfilamentous mutant. PLoS Genet. 2014;10(12):e1004824 Epub 2014/12/05. 10.1371/journal.pgen.1004824 25474009 PMC4256171

[41] Hu G, Chen SH, Qiu J, Bennett JE, Myers TG, Williamson PR. Microevolution during serial mouse passage demonstrates *FRE3* as a virulence adaptation gene in *Cryptococcus neoformans*. MBio. 2014;5(2):e00941–14. Epub 2014/04/03. 10.1128/mBio.00941-14 24692633 PMC3977352

[42] Wilson D, Citiulo F, Hube B. Zinc exploitation by pathogenic fungi. PLoS Pathog. 2012;8(12):e1003034 Epub 2013/01/12. 10.1371/journal.ppat.1003034 23308062 PMC3534374

[43] Ma Q, Ola M, Iracane E, Butler G. Susceptibility to Medium-Chain Fatty Acids Is Associated with Trisomy of Chromosome 7 in *Candida albicans*. mSphere. 2019;4(3). Epub 2019/06/28. 10.1128/mSphere.00402-19 31243082 PMC6595153

[44] Cheng S, Clancy CJ, Zhang Z, Hao B, Wang W, Iczkowski KA, et al Uncoupling of oxidative phosphorylation enables *Candida albicans* to resist killing by phagocytes and persist in tissue. Cell Microbiol. 2007;9(2):492–501. Epub 2006/09/22. 10.1111/j.1462-5822.2006.00805.x . 16987332

[45] Kowalski CH, Beattie SR, Fuller KK, McGurk EA, Tang YW, Hohl TM, et al Heterogeneity among Isolates Reveals that Fitness in Low Oxygen Correlates with *Aspergillus fumigatus Virulence*. MBio. 2016;7(5). Epub 2016/09/22. 10.1128/mBio.01515-16 PubMed PMID: 27651366; PubMed Central PMCID: PMC5040115. PMC5040115 27651366

[46] Kowalski CH, Kerkaert JD, Liu KW, Bond MC, Hartmann R, Nadell CD, et al Fungal biofilm morphology impacts hypoxia fitness and disease progression. Nat Microbiol. 2019 Epub 2019/09/25. 10.1038/s41564-019-0558-7 . 31548684 PMC7396965

[47] Sheltzer JM, Blank HM, Pfau SJ, Tange Y, George BM, Humpton TJ, et al Aneuploidy drives genomic instability in yeast. Science. 2011;333(6045):1026–30. Epub 2011/08/20. 10.1126/science.1206412 21852501 PMC3278960

[48] Torres EM, Sokolsky T, Tucker CM, Chan LY, Boselli M, Dunham MJ, et al Effects of aneuploidy on cellular physiology and cell division in haploid yeast. Science. 2007;317(5840):916–24. Epub 2007/08/19. 10.1126/science.1142210 . 17702937

[49] Tsai HJ, Nelliat A. A Double-Edged Sword: Aneuploidy is a Prevalent Strategy in Fungal Adaptation. Genes, 2019;10(10). 10.3390/genes10100787 PubMed Central PMCID: PMC6826469 31658789 PMC6826469

[50] Gilchrist C, Stelkens R. Aneuploidy in yeast: Segregation error or adaptation mechanism? Yeast. 2019 Epub 2019/06/15. 10.1002/yea.3427 31199875 PMC6772139

[51] Arita GS, Meneguello JE, Sakita KM, Faria DR, Pilau EJ, Ghiraldi-Lopes LD, et al Serial Systemic *Candida albicans* Infection Highlighted by Proteomics. Front Cell Infect Microbiol. 2019;9:230 Epub 2019/07/12. 10.3389/fcimb.2019.00230 31293987 PMC6606696

